# Adverse childhood experiences and adult diet quality

**DOI:** 10.1017/jns.2021.85

**Published:** 2021-10-29

**Authors:** Sydney R. Aquilina, Martha J. Shrubsole, Julia Butt, Maureen Sanderson, David G. Schlundt, Mekeila C. Cook, Meira Epplein

**Affiliations:** 1 Trinity College of Arts & Sciences, Duke University, Durham, NC, USA; 2 Division of Epidemiology, Department of Medicine, Vanderbilt-Ingram Cancer Center, Vanderbilt University School of Medicine, Nashville, TN, USA; 3 Infections and Cancer Epidemiology, German Cancer Research Center, Heidelberg, Germany; 4 Department of Family and Community Medicine, Meharry Medical College, Nashville, TN, USA; 5 Department of Psychology, Vanderbilt University, Nashville, TN, USA; 6 Division of Public Health Practice, Meharry Medical College, Nashville, TN, USA; 7 Department of Population Health Sciences, Duke University and Cancer Risk, Detection, and Interception Program, Duke Cancer Institute, Durham, NC, USA

**Keywords:** Adverse childhood experiences, Child maltreatment, Childhood trauma, Diet quality, Dietary components, Healthy Eating Index, Household dysfunction, Low-income population

## Abstract

Childhood trauma is strongly associated with poor health outcomes. Although many studies have found associations between adverse childhood experiences (ACEs), a well-established indicator of childhood trauma and diet-related health outcomes, few have explored the relationship between ACEs and diet quality, despite growing literature in epidemiology and neurobiology suggesting that childhood trauma has an important but poorly understood relationship with diet. Thus, we performed a cross-sectional study of the association of ACEs and adult diet quality in the Southern Community Cohort Study, a largely low-income and racially diverse population in the southeastern United States. We used ordinal logistic regression to estimate the association of ACEs with the Healthy Eating Index-2010 (HEI-10) score among 30 854 adults aged 40–79 enrolled from 2002 to 2009. Having experienced any ACE was associated with higher odds of worse HEI-10 among all (odds ratio (OR) 1⋅22; 95 % confidence interval (CI) 1⋅17, 1⋅27), and for all race–sex groups, and remained significant after adjustment for adult income. The increasing number of ACEs was also associated with increasing odds of a worse HEI-10 (OR for 4+ ACEs: 1⋅34; 95 % CI 1⋅27, 1⋅42). The association with worse HEI-10 score was especially strong for ACEs in the household dysfunction category, including having a family member in prison (OR 1⋅34; 95 % CI 1⋅25, 1⋅42) and parents divorced (OR 1⋅25; 95 % CI 1⋅20, 1⋅31). In summary, ACEs are associated with poor adult diet quality, independent of race, sex and adult income. Research is needed to explore whether trauma intervention strategies can impact adult diet quality.

## Introduction

Adverse childhood experiences (ACEs) include a wide breadth of exposures related to abuse, household dysfunction and neglect. In the original ACE study by CDC-Kaiser, these exposures were assessed in two waves through an extensive multipart health questionnaire totalling sixty-eight questions in the final wave^(1)^. Subsequent studies have condensed the original questionnaire into ten questions, representing: emotional abuse; physical abuse; sexual abuse; emotional neglect; physical neglect; parents divorced; mother abused; live with alcohol or drug use; live with depression or suicide and family member in prison^(1–4)^.

ACEs are now understood to be both common, affecting approximately two-thirds of the general population, and pervasive, increasing risk of many unhealthy behaviours and chronic diseases^(1,5–7)^. For instance, people with four or more ACEs were twice as likely to smoke, seven times more likely to be an alcoholic and twelve times more likely to have attempted suicide in comparison to those with three or fewer ACEs in the original ACE study. ACEs were associated with many chronic health conditions including asthma, cardiovascular disease, diabetes and cancer^(1,8)^. In addition, studies suggest that the effects of ACEs are cumulative. For example, a study by Merrick *et al.* found ACEs to have a cumulative effect (across 1, 2–3 and 4+ ACEs) on the odds of chronic conditions, depression, health risk behaviours (smoking, heavy drinking) and socioeconomic challenges^(9)^. Among racial minorities and individuals with lower socioeconomic status (SES), associations have been found to be even stronger^(10–13)^.

The Healthy Eating Index-2010 (HEI-10) is a valid and reliable measure of diet quality developed by the United States Department of Agriculture (USDA) and U.S. Department of Health and Human Services (HHS) to reflect the Dietary Guidelines for Americans^(14,15)^. However, neither the HEI-10 nor any comprehensive food frequency questionnaire (FFQ) has been used to evaluate the impact of ACEs on adult diet, despite growing literature in epidemiology and neurobiology suggesting that childhood trauma has an important but poorly understood relationship with diet^(16)^.

For instance, associations have been found between ACEs and many diet-related conditions such as obesity, binge-eating disorder, food addiction, irritable bowel syndrome, inflammatory bowel disease, bulimia nervosa, anorexia nervosa, elevated cortisol levels, pro-inflammatory gut microbiota and more general dysregulation of the immune and endocrine systems^(17–27)^. Additionally, a recent study by Schuler *et al.* found cumulative and individual ACEs to predict poor diet quality among children^(28)^. By establishing evidence of an association of ACEs with poor diet quality in childhood, Schuler *et al.*'s findings also suggest the possibility of ACE-induced poor diet quality in adulthood through a continuation of poor childhood diet. Given the complexity of the impact of childhood trauma on both brain structure (e.g. size of prefrontal cortex) and function (e.g. dysregulation of the hypothalamic–pituitary–adrenal (HPA) axis), the multi-system involvement of these variables within the human body, and the contextual importance of environmental factors for any such variables or systems, there are many pathways which could potentially result in an ACE–adult diet quality (as represented by the HEI-10) association^(19,29–34)^.

In the present study, we sought to evaluate the association of ACEs and adult diet quality assessed using an FFQ in the Southern Community Cohort Study (SCCS), a prospective study of approximately 85 000 adults over age 40 in the southeastern United States established to study cancer and other health outcomes, particularly in underserved populations^(35)^. Previously, studies of SCCS participants have found ACEs to be associated with adult cancer risk behaviours, greater chronic disease burden and higher adult health care utilisation. These studies also suggest that there may be race and sex differences for ACEs that have not been well explored^(36,37)^. Another study conducted in the SCCS used the HEI-10, finding better diet quality to be associated with a lower risk of death for all-cause mortality^(38)^. These results suggest that the HEI-10 derived from an FFQ is a good proxy for diet quality in this population.

Ultimately, the present study aims to broaden our understanding of the associations of childhood trauma and adult health. More specifically, the present study explores whether having traumatic experiences during childhood significantly increases odds of poor diet as an adult, and whether these associations differ by race or sex, among participants of the SCCS. Our *a priori* hypothesis is that ACEs are associated with lower quality of adult diet, and that this association might be particularly impactful among racially marginalised communities.

## Methods

### Study design and participants

Data were collected from the SCCS, a multi-year prospective cohort of approximately 85 000 adults from 12 southeastern states in the US enrolled between ages 40–79 in the years 2002–9, primarily from community health clinics that serve the under- and uninsured^(35)^. We included participants who (1) completed the FFQ from the baseline at enrolment, such that HEI-10 could be calculated and (2) completed the ACE survey from the second follow-up questionnaire administered in 2012^(35)^.

Of the 84 508 total current SCCS participants, those with insufficient completion of the FFQ (>10 food items left blank) and implausible energy intakes (<600 or >8000 kcal/d) were excluded (*n* 6042). Additionally, since numbers were small for racial groups other than Black or White (*n* 4123), we did not include them in the present analysis. Of these 74 343, 36 569 completed the second follow-up questionnaire, and were more likely to be older, female, white and have higher SES compared with the total population, while still generally reflecting the overall low SES SCCS population. Of these participants who returned for the second follow-up, 84⋅4 % completed the ACE questionnaire in full, such that only 15⋅6 % (*n* 5715) were excluded because they were missing at least one of the ACE questions. Thus, we performed a retrospective cohort analysis including a total of 30 854 SCCS participants (18 769 Blacks; 12 085 Whites). Analyses were conducted by self-reported sex and race among all and in the following groups: Black females (*n* 12 576); Black males (*n* 6193); White females (*n* 7704); White males (*n* 4381). Importantly, the relationship between ACEs and adult HEI-10 can be assumed to be temporal, as ACEs are acquired during childhood and adult HEI-10 was the outcome measured. Due to the high cost of a healthy diet, which could have a disproportionate impact on the population by ACE score, we secondarily included adult household income as a variable in the model to estimate the ACE-HEI association above and beyond the association with income level.

### Study instruments and assessment of variables

The original ACE questionnaire described earlier has been well validated as a reliable measure of childhood trauma^(1–4)^. The usage of condensed versions has also been found to be valid and reliable^(39,40)^. Thus, a modified version of the original ACE questionnaire was used in the present study with only ten questions, each reflecting one of the ten identified ACEs of emotional abuse, physical abuse, sexual abuse, emotional neglect, physical neglect, parents divorced, mother physically abused, lived with an alcohol or drug abuser, had a household member who was mentally ill or attempted suicide, and had a household member go to prison. Results from this abbreviated ACE questionnaire have been found to be associated with adult cancer risk behaviours and health care utilisation in the SCCS population^(36,37)^.

The FFQ created and utilised by the SCCS (as previously described^(41)^) was empirically designed to account for race and geographic region using the National Health and Nutrition Examination Survey (NHANES) and Continuing Survey of Food Intakes by Individuals (CSFII) 24-hour recall databases^(42)^. From the resulting FFQ variables, the HEI-10 score was calculated^(38)^. As discussed earlier, the HEI-10 is a valid and reliable measure of diet quality developed by the USDA and HHS to reflect the Dietary Guidelines for Americans^(14,15)^. There are twelve components included in the HEI-10 score (scaled from 0 to 100), which are added together for the total HEI-10 score, and included: total fruit, whole fruit, whole grains, total dairy products, total vegetables, greens and beans, total protein foods, seafood and plant proteins, fatty acids, sodium, solid fat/alcohol/added sugar and refined grains. Depending on the component, scoring is based on adequacy or moderation, including fatty acids which is scored using an adequacy ratio of poly- and monounsaturated to saturated fat^(14)^.

For the population under study, we first examined the associations of socio-demographic and lifestyle variables with the exposure of interest, ACEs. More specifically, the variables included were age (as a continuous variable), BMI (in three categories: <25, 25–29⋅9, ≥30), education (in three categories: less than high school, high school or General Education Diploma (GED), more than high school), household income (in four categories: <$15 000, $15 000–<$25 000, $25 000–<$50 000, ≥$50 000), enrolment source (in two categories: community health centre, general population), marital status (in four categories: married or living as married, separated or divorced, widowed, single), neighbourhood deprivation index (in quartiles), smoking status (in four categories: never, former, current and <20 cigarettes per day, current and ≥20 cigarettes per day) and alcohol intake (in four categories: never or rarely, 1–3 times per month, 1–4 times per week, daily or near daily).

### Statistical analysis

In order to explore the distribution of the socio-demographic and lifestyle variables by ACEs, we compared the mean of the continuous variable (age) and the frequencies of the categorical variables (all other variables, as listed above) across the five conventional ACE score categories, namely 0, 1, 2, 3 and 4 or more reported ACEs. Pearson *χ*^2^ tests were utilised to determine the association of each respective categorical variable with the ACE score, and within each race/sex group. For age, student's *t* tests were used.

Similarly, we examined the distribution of the same baseline variables by the outcome HEI-10, in five categories of quintiles, with the fifth and highest quintile being the least healthy diet and the first and lowest quintile being the most healthy diet (this is the reverse of conventional HEI-10 scoring, but in line with the directionality of ACE scoring). Quintile categories were determined from the general population of participants and applied to each of the four race/sex groups. As in the baseline analysis by ACEs, associations between HEI-10 quintiles and each respective baseline variable were assessed using Pearson *χ*^2^ tests and student's *t* tests for the continuous variable of age.

For the main analyses, ordinal logistic regression using proportional odds models were used to examine the association of ACEs with the outcome of HEI-10 in the same categories of quintiles. We also explored the association with a nominal polytomous model, with five potential outcomes (HEI-10 quintiles 1–5), but our analyses suggested that it was appropriate to use a simplified model with HEI-10 as one ordered categorical variable, with increasing quintile as the outcome. We calculated odds ratios (ORs) and 95 % confidence intervals (CIs) for increasing HEI-10 score (as a marker of worse diet quality) with the exposure of ACEs in four ways: by any ACE; by number of ACEs (in categories of 0, 1, 2, 3 and 4+); by ACE category (abuse, neglect, household dysfunction); with each of the ten individual ACEs. Having no ACEs at all, no ACEs for that category, and not having the individual ACE in question were used for reference.

Since ACEs occur in childhood, and all other data used in the present study, with the exception of race and sex, pertains to adulthood, variables associated with both ACEs and HEI-10 would not confound the association (as they would not affect the main exposure, of ACEs), and thus are presented to describe the population, but are not included as variables in the main results models.

As a secondary analysis, we included adult income as a variable, to determine the ACEs and HEI-10 association above and beyond the association with adult income, considering the higher cost of a high-quality diet. To do this, we used the same proportional odds models as discussed above, but then adjusted for adult annual household income in four categories: <$15 000; $15 000–$24 999; $25 000–$49 999; ≥$50 000 (using <$15 000 as the reference). As education was not associated with ACEs among all, and we found adjusting for education to have no effect on our ORs beyond adjustment for household income, we decided not to include it in our final analyses.

To further our understanding of the dietary contributors to the ACEs and HEI-10 association, we performed proportional odds logistic regression to calculate odds ratios of the exposure of any ACE and each HEI-10 component as an outcome. Again, we calculated ORs within each race/sex group and for all (adjusting for race and sex), and secondarily adjusting for annual household income.

## Results

ACEs varied significantly with most adult socio-demographic and lifestyle variables (Table 1). In every race/sex group (Black females, Black males, White females and White males), more ACEs were reported by individuals who were younger, had lower household incomes were enrolled at a community health centre (*v.* the general population) were divorced or had never been married, and were smokers and drinkers. Among White females and White males only, a greater number of ACEs was also reported by obese individuals.

**Table 1.** Distribution of socio-demographic and lifestyle variables by number of adverse childhood experiences (ACEs), stratified by race and sex

	All participants (*n* 30 854)					Black Females (*n* 12 576)					Black Males (*n* 6193)					White Females (*n* 7704)					White Males (*n* 4381)				
	ACE Score					ACE Score					ACE Score					ACE Score					ACE Score				
ACE Score	0	1	2	3	4+	0	1	2	3	4+	0	1	2	3	4+	0	1	2	3	4+	0	1	2	3	4+
*n*	13 150	6877	3507	2125	5195	5188	3071	1467	864	1986	2707	1520	756	401	809	3030	1431	866	600	1777	2225	855	418	260	623
(%)	(43)	(22)	(11)	(7)	(17)	(41)	(24)	(12)	(7)	(16)	(44)	(25)	(12)	(6)	(13)	(39)	(19)	(11)	(8)	(23)	(51)	(20)	(10)	(6)	(14)
*Socio-demographic*																									
Age in years, mea*n* (sd)^1^^,^^2^^,^^3^^,^^4^^,^^5^	55 (9)	53 (8)	52 (8)	51 (8)	50 (7)	53 (9)	52 (8)	51 (8)	50 (8)	49 (7)	53 (8)	51 (8)	50 (8)	49 (7)	49 (6)	56 (9)	54 (9)	54 (8)	53 (8)	51 (7)	58 (9)	56 (9)	55 (8)	54 (9)	52 (8)
Body mass index in kg/m^2^ (%)^1^^,^^4^^,^^5^																									
<25⋅0	23	22	23	23	22	14	14	15	17	15	29	31	33	35	33	31	26	27	22	23	25	24	21	22	24
25⋅0–29⋅9	32	30	29	30	28	26	27	25	25	24	37	36	34	36	36	29	25	25	28	26	44	40	42	40	36
≥30⋅0	45	48	48	48	50	60	58	60	58	61	34	34	33	29	32	40	49	48	50	51	31	36	37	38	40
Education (%)^2^^,^^5^																									
Less than High School	20	22	21	21	22	24	25	24	22	24	27	24	23	28	25	17	16	17	16	18	10	14	13	14	20
High School or GED	31	31	30	31	31	34	32	31	30	28	34	34	35	33	36	32	31	29	32	33	22	23	22	27	30
More than High School	48	48	49	49	47	42	43	45	47	47	40	42	42	39	39	51	53	54	53	49	68	63	66	60	50
Household Income (%)^1^^,^^2^^,^^3^^,^^4^^,^^5^																									
<15 000	41	45	46	50	53	52	53	52	56	58	48	50	50	59	58	33	36	40	43	49	18	25	27	32	43
15 000−<25 000	21	21	21	21	21	25	24	24	23	22	22	20	23	23	22	19	18	18	19	21	12	14	17	16	17
25 000−<50 000	19	18	18	16	16	15	16	17	15	14	17	18	17	12	14	23	21	21	18	18	22	21	21	21	18
≥50 000	20	16	15	13	10	7⋅4	7⋅1	7⋅2	6⋅6	5⋅6	14	12	10	6⋅6	6⋅6	26	25	21	19	12	49	39	35	30	22
Enrolment source^1^^,^^3^^,^^4^^,^^5^																									
Community health centre	73	79	77	79	82	87	88	86	86	86	81	83	83	86	87	69	71	70	76	80	38	50	49	56	68
General population	27	21	23	21	18	13	12	14	14	14	19	17	17	14	13	31	29	30	24	20	62	50	51	44	32
Marital Status (%)^1^^,^^2^^,^^3^^,^^4^^,^^5^																									
Married, Living as Married	48	41	40	38	36	32	30	30	28	26	48	40	36	31	29	58	52	49	48	44	76	69	66	59	52
Separated, Divorced	25	31	33	34	38	32	34	36	38	40	26	32	31	34	36	22	29	34	35	40	14	19	20	20	32
Widowed	10	9⋅0	7⋅8	7⋅9	6⋅9	15	13	10	10	8⋅4	3⋅1	2⋅7	4⋅0	3⋅3	2⋅9	15	11	10	10	8⋅4	2⋅8	2⋅0	3⋅0	2⋅0	2⋅8
Single, Never been married	16	19	19	20	19	21	23	24	24	26	23	25	28	32	33	5⋅8	7⋅9	7⋅0	6⋅6	8⋅0	6⋅9	10	12	18	14
Neighbourhood Deprivation Index (%)^1^^,^^2^^,^^3^^,^^4^^,^^5^																									
Quartile 1 (least deprived)	15	13	13	13	13	6⋅3	6⋅2	6⋅7	6⋅6	6⋅3	7⋅5	6⋅7	6⋅5	4⋅0	7⋅2	26	24	22	22	20	32	31	30	27	22
Quartile 2	18	17	19	18	20	11	11	13	11	12	10	10	12	11	8⋅7	29	30	29	30	31	28	28	31	27	27
Quartile 3	23	21	21	23	23	21	18	16	20	20	19	17	17	15	15	30	29	31	31	30	25	25	23	26	26
Quartile 4 (most deprived)	43	49	47	46	44	62	65	64	63	62	63	66	65	70	69	15	16	18	17	20	14	16	17	21	24
*Lifestyle*																									
Smoker (%)^1^^,^^2^^,^^3^^,^^4^^,^^5^																									
Never	48	43	38	36	31	58	51	46	43	38	29	27	25	21	19	53	47	42	39	31	39	33	29	28	24
Former	27	26	27	26	25	19	21	21	22	22	28	26	24	22	22	27	29	31	29	28	43	40	46	41	34
Current < 20 cpd	18	21	23	24	26	19	22	26	26	30	32	36	38	41	43	10	10	10	14	18	5⋅7	8⋅3	8⋅5	13	13
Current ≥ 20 cpd	8⋅3	10	12	14	18	4⋅5	5⋅8	7⋅5	9⋅2	10	10	11	13	17	17	10	14	16	18	24	12	19	16	19	29
Alcohol (%)^1^^,^^2^^,^^3^^,^^4^^,^^5^																									
Never/Rarely	35	31	28	26	25	47	41	36	32	32	13	12	11	9⋅0	7⋅4	46	38	35	34	29	18	14	13	14	12
1–3 times per month	11	12	12	10	10	13	14	14	13	12	7⋅4	8⋅6	8⋅7	7⋅4	7⋅8	12	14	12	12	11	7⋅7	8⋅6	7⋅4	5⋅2	7⋅0
1–4 times per week	33	33	35	34	32	29	31	34	33	31	41	38	38	35	35	29	30	34	35	33	36	36	35	37	31
Daily/Nearly Daily	22	24	26	29	32	11	14	16	23	24	39	41	42	49	50	13	18	20	19	27	39	41	45	44	49

Abbreviations: ACE, adverse childhood experiences; cpd, cigarettes per day.

1 Significant at *P* < 0⋅05 by ACE score for all.

2 Significant at *P* < 0⋅05 by ACE score for Black females.

3 Significant at *P* < 0⋅05 by ACE score for Black males.

4 Significant at *P* < 0⋅05 by ACE score for White females.

5 Significant at *P* < 0⋅05 by ACE score for White males.

The Healthy Eating Index-10 was also strongly associated with the adult socio-demographic and lifestyle variables (Table 2). In fact, every socio-demographic and lifestyle variable was associated with HEI-10 among all and in every race/sex group. In general, the trends found were similar to those in Table 1 with ACEs. For instance, a worse HEI-10 (represented by the highest quintile in the present study) was more common among younger individuals, people with lower household income, participants recruited from the community health centres (*v.* the general population), current smokers, frequent drinkers, and those who were divorced or never married, except for Black females, among whom divorced females were more likely to have a better HEI-10. While higher BMI was associated with worse HEI-10 among Whites, higher BMI was associated with better HEI-10 among Blacks. Unlike with ACEs, education was associated consistently and strongly with HEI-10, such that individuals with high school education or less were more likely to have worse HEI-10 across the race/sex groups than individuals with more than a high school education. Furthermore, current smoking, especially greater than 20 cigarettes per day, and more frequent alcohol intake were associated with worse HEI-10 in every race/sex group.

**Table 2.** Distribution of socio-demographic and lifestyle variables by Healthy Eating Index (HEI) quintile, stratified by race and sex

	All participants (*n* 30 854)					Black Females (*n* 12 576)					Black Males (*n* 6193)					White Females (*n* 7704)					White Males (*n* 4381)				
	HEI quintile					HEI quintile					HEI quintile					HEI quintile					HEI quintile				
HEI quintile	1	2	3	4	5	1	2	3	4	5	1	2	3	4	5	1	2	3	4	5	1	2	3	4	5
*n*	6171	6170	6172	6170	6171	2796	2761	2593	2381	2045	753	1054	1279	1483	1624	1795	1593	1471	1388	1457	827	762	829	918	1045
(%)	(20)	(20)	(20)	(20)	(20)	(22)	(22)	(21)	(19)	(16)	(12)	(17)	(21)	(24)	(26)	(23)	(21)	(19)	(18)	(19)	(19)	(17)	(19)	(21)	(24)
*Socio-demographic*																									
Age in years, mea*n* (sd)^1^^,^^2^^,^^3^^,^^4^^,^^5^	56 (9)	54 (9)	53 (8)	51 (8)	50 (8)	55 (9)	53 (8)	51 (8)	50 (8)	49 (7)	55 (9)	53 (8)	51 (7)	50 (7)	50 (7)	58 (9)	56 (9)	54 (8)	53 (8)	51 (8)	59 (8)	58 (9)	56 (9)	55 (8)	53 (8)
Body mass index in kg/m^2^ (%)^1^^,^^2^^,^^3^^,^^4^^,^^5^																									
<25⋅0	22	19	22	23	26	12	12	15	16	18	17	26	30	34	38	34	25	25	24	26	29	24	21	22	25
25⋅0–29⋅9	33	30	31	30	29	27	27	26	25	22	42	35	38	34	34	30	27	25	26	26	49	42	42	39	37
≥30⋅0	46	51	48	47	45	61	61	58	59	59	41	39	32	32	28	36	49	49	50	48	22	34	37	38	38
Education (%)^1^^,^^2^^,^^3^^,^^4^^,^^5^																									
Less than High School	13	17	22	25	28	17	21	27	29	29	16	23	23	28	31	9⋅7	13	16	21	27	5⋅3	5⋅7	12	17	20
High School or GED	23	29	32	34	38	26	32	31	35	37	22	30	36	37	39	26	30	34	34	38	11	17	22	27	37
More than High School	63	53	47	41	35	57	47	42	36	34	62	47	41	35	31	65	57	50	46	35	84	77	66	57	43
Household Income (%)^1^^,^^2^^,^^3^^,^^4^^,^^5^																									
<15 000	33	41	47	50	56	43	50	56	59	63	30	43	50	54	62	29	35	39	43	50	11	15	24	29	39
15 000−<25 000	19	21	21	22	21	23	25	23	24	23	21	20	23	24	21	16	18	20	21	21	9⋅9	11	12	16	18
25 000−<50 000	22	19	18	16	14	21	17	15	13	10	23	20	17	15	13	23	22	21	19	18	20	22	21	21	21
≥50 000	26	19	15	12	8⋅8	12	7⋅8	5⋅7	4⋅1	3⋅3	26	17	11	6⋅8	4⋅8	32	25	20	17	11	59	52	42	34	22
Enrolment source^1^^,^^2^^,^^3^^,^^4^^,^^5^																									
Community health centre	69	75	78	80	83	82	87	88	89	91	69	78	82	86	90	66	70	73	75	80	31	37	45	53	62
General population	31	25	23	20	17	18	13	12	11	9⋅1	31	22	18	14	10	34	30	27	25	20	69	63	55	47	38
Marital Status (%)^1^^,^^2^^,^^3^^,^^4^^,^^5^																									
Married, Living as Married	47	45	43	41	39	31	32	30	28	28	53	49	41	38	33	55	51	52	51	50	78	77	72	67	58
Separated, Divorced	29	29	30	31	32	37	34	34	35	33	26	26	30	32	33	26	29	30	30	34	13	14	16	20	26
Widowed	12	10	9⋅1	7⋅9	6⋅5	15	14	12	11	10	4⋅2	2⋅6	3⋅7	2⋅8	2⋅5	13	13	12	11	9⋅0	3⋅0	2⋅4	2⋅6	2⋅0	3⋅0
Single, Never been married	13	16	18	20	22	18	20	23	26	29	16	23	26	28	32	6⋅1	7⋅6	6⋅0	7⋅8	7⋅0	6⋅3	6⋅3	9⋅6	11	13
Neighbourhood Deprivation Index (%)^1^^,^^2^^,^^3^^,^^4^^,^^5^																									
Quartile 1 (least deprived)	21	16	13	11	8⋅5	9⋅8	6⋅4	4⋅9	5⋅6	4⋅3	13	9⋅3	6⋅9	5⋅4	4⋅1	31	27	23	20	13	43	36	33	24	17
Quartile 2	20	18	17	17	18	14	12	11	10	9⋅3	15	11	9⋅7	9⋅4	8⋅9	29	30	29	31	31	28	27	28	28	29
Quartile 3	22	23	22	22	23	20	21	19	18	18	22	19	18	17	16	26	28	31	31	34	19	24	25	28	29
Quartile 4 (most deprived)	37	43	47	49	51	56	61	65	67	69	50	61	66	68	71	13	15	17	18	22	10	12	15	20	24
*Lifestyle*																									
Smoker (%)^1^^,^^2^^,^^3^^,^^4^^,^^5^																									
Never	53	46	41	36	33	59	52	49	45	44	39	29	25	24	22	52	50	44	38	35	44	41	34	28	26
Former	33	29	26	24	19	26	22	20	17	14	41	32	26	22	18	35	31	27	26	19	49	45	45	40	32
Current < 20 cpd	11	18	22	26	27	13	21	24	29	31	18	29	38	40	42	8⋅3	10	13	16	16	2⋅7	5⋅9	8⋅4	9⋅0	12
Current ≥ 20 cpd	3⋅2	6⋅8	11	15	20	2⋅3	4⋅1	6⋅7	8⋅8	12	2⋅3	9⋅6	11	14	17	4⋅7	9⋅0	16	20	29	4⋅2	8⋅3	13	23	30
Alcohol (%)^1^^,^^2^^,^^3^^,^^4^^,^^5^																									
Never/Rarely	37	34	30	27	25	48	43	41	36	34	18	15	11	11	8⋅4	39	40	37	38	39	14	18	17	15	15
1–3 times per month	12	12	11	11	10	13	14	13	13	14	10	8⋅9	7⋅1	8⋅7	6⋅0	12	13	11	13	12	8⋅0	9⋅2	8⋅3	5⋅8	7⋅1
1–6 times per week	34	33	34	33	31	30	31	31	31	32	42	40	41	39	34	33	32	33	29	28	42	34	36	36	30
Daily	18	21	25	29	34	9⋅4	13	16	20	21	30	36	41	41	52	17	16	18	20	22	36	40	39	43	47

Abbreviations: cpd, cigarettes per day; HEI, Healthy Eating Index.

1 Significant at *P* < 0⋅05 by HEI score for all.

2 Significant at *P* < 0⋅05 by HEI score for Black females.

3 Significant at *P* < 0⋅05 by HEI score for Black males.

4 Significant at *P* < 0⋅05 by HEI score for White females.

5 Significant at *P* < 0⋅05 by HEI score for White males.

For the main analyses, being exposed to any childhood trauma (as defined by ACE screening) was associated with an increased odds of a worse HEI-10, compared to having no ACE (OR 1⋅22; 95 % CI 1⋅17, 1⋅27) (Table 3). This finding was consistent across all race/sex groups, and remained significant after adjusting for adult household income in the model. By including household income as a variable in the model, we estimated the effect of ACEs on diet quality above and beyond the association with adult household income. Thus, our adjusted results show a conservative estimate of the ACE-HEI association and suggest that there is a pathway from ACEs to diet quality outside of the association with income. Similarly, we found an increasing odds of worse HEI-10 with the increasing number of ACEs among all study participants and in every race/sex group before and after adjustment for income. Individuals with four or more ACEs have a 34 % increased relative odds of a worse HEI-10 compared to individuals with no ACEs (95 % CI 1⋅27, 1⋅42). By ACE category, associations were strongest for household dysfunction (all without income adjustment, OR 1⋅24; 95 % CI 1⋅19, 1⋅29; all with income adjustment, OR 1⋅16; 95 % CI 1⋅11, 1⋅20). For abuse, ORs were statistically significant without income adjustment and after adjustment (OR 1⋅12; 95 % CI 1⋅07, 1⋅17 and OR 1⋅06; 95 % CI 1⋅01, 1⋅11, respectively). Associations for neglect were stronger than for abuse, although not as strong as for household dysfunction, with ORs reaching 1⋅20 (95 % CI 1⋅15, 1⋅27) and 1⋅07 (95 % CI 1⋅02, 1⋅13) for before and after income adjustment. However, in the specific race/sex-specific groupings, neglect was significant after adjustment for income only among Black females (OR 1⋅09; 95 % CI 1⋅00, 1⋅18, respectively). Most of the ten individual ACEs were associated with HEI-10 both before and after adjustment for income. The only exceptions were for emotional abuse and physical neglect, which were significant before but not after income adjustment. The three strongest individual ACEs were significant before and after income adjustment among all and for three of the four race/sex groups. They were all household dysfunction ACEs, namely having a family member in prison (OR 1⋅34; 95 % CI 1⋅25, 1⋅42), having divorced or separated parents (OR 1⋅25; 95 % CI 1⋅20, 1⋅31), and living with alcohol or drug abuse (OR 1⋅19; 95 % CI 1⋅14, 1⋅25).

**Table 3.** Association of ACEs with a lower Healthy Eating Index, by race–sex groupings

	All (*n* 30 854)			Black Females (*n* 12 576)			Black Males (*n* 6193)			White Females (*n* 7704)			White Males (*n* 4381)		
	*n* (%)	Race and sex adjusted OR	Race, sex and income adjusted OR^1^	*n* (%)	Unadjusted OR	Income adjusted OR^1^	*n* (%)	Unadjusted OR	Income adjusted OR^1^	*n* (%)	Unadjusted OR	Income adjusted OR^1^	*n* (%)	Unadjusted OR	Income adjusted OR^1^
By any ACE															
None	13 150 (43)	1⋅00 (ref)	1⋅00 (ref)	5188 (41)	1⋅00 (ref)	1⋅00 (ref)	2707 (44)	1⋅00 (ref)	1⋅00 (ref)	3030 (39)	1⋅00 (ref)	1⋅00 (ref)	2225 (51)	1⋅00 (ref)	1⋅00 (ref)
Any	17 704 (57)	**1⋅22 (1⋅17, 1⋅27)**	**1⋅15 (1⋅10, 1⋅19)**	7388 (59)	**1⋅13 (1⋅06,1⋅21)**	**1⋅13 (1⋅06, 1⋅20)**	3486 (56)	**1⋅19 (1⋅09, 1⋅30)**	**1⋅12 (1⋅03, 1⋅23)**	4674 (61)	**1⋅22 (1⋅13, 1⋅32)**	**1⋅13 (1⋅04, 1⋅23)**	2156 (49)	**1⋅51 (1⋅36, 1⋅67)**	**1⋅24 (1⋅11, 1⋅38)**
By number of ACEs															
0 ACEs	13 150 (43)	1⋅00 (ref)	1⋅00 (ref)	5188 (41)	1⋅00 (ref)	1⋅00 (ref)	2707 (44)	1⋅00 (ref)	1⋅00 (ref)	3030 (39)	1⋅00 (ref)	1⋅00 (ref)	2225 (51)	1⋅00 (ref)	1⋅00 (ref)
1 ACE	6877 (22)	**1⋅15 (1⋅09, 1⋅21)**	**1⋅13 (1⋅07, 1⋅19)**	3070 (24)	**1⋅11 (1⋅02, 1⋅20)**	**1⋅11 (1⋅02, 1⋅20)**	1520 (25)	**1⋅12 (1⋅00, 1⋅25)**	**1⋅12 (1⋅00, 1⋅26)**	1430 (19)	**1⋅13 (1⋅01, 1⋅26)**	1⋅11 (0⋅99, 1⋅24)	855 (20)	**1⋅31 (1⋅14, 1⋅50)**	**1⋅18 (1⋅03, 1⋅36)**
2 ACEs	3507 (11)	**1⋅17 (1⋅09, 1⋅25)**	**1⋅12 (1⋅05, 1⋅20)**	1467 (12)	**1⋅11 (1⋅01, 1⋅23)**	**1⋅13 (1⋅02, 1⋅26)**	756 (12)	1⋅14 (0⋅99, 1⋅32)	1⋅10 (0⋅95, 1⋅27)	866 (11)	1⋅13 (0⋅99, 1⋅29)	1⋅07 (0⋅93, 1⋅22)	418 (10)	**1⋅38 (1⋅15, 1⋅67)**	1⋅17 (0⋅97, 1⋅41)
3 ACEs	2125 (6⋅9)	**1⋅27 (1⋅17, 1⋅38)**	**1⋅17 (1⋅08, 1⋅27)**	864 (6⋅9)	**1⋅19 (1⋅05, 1⋅36)**	**1⋅19 (1⋅05, 1⋅36)**	401 (6⋅5)	**1⋅28 (1⋅06, 1⋅54)**	1⋅10 (0⋅91, 1⋅33)	600 (7⋅8)	**1⋅22 (1⋅05, 1⋅43)**	1⋅13 (0⋅97, 1⋅32)	260 (5⋅9)	**1⋅53 (1⋅22, 1⋅92)**	1⋅23 (0⋅98, 1⋅56)
4+ ACEs	5195 (17)	**1⋅34 (1⋅27, 1⋅42)**	**1⋅18 (1⋅11, 1⋅25)**	1986 (16)	**1⋅16 (1⋅05, 1⋅27)**	**1⋅11 (1⋅02, 1⋅22)**	809 (13)	**1⋅31 (1⋅14, 1⋅51)**	**1⋅16 (1⋅01, 1⋅34)**	1777 (23)	**1⋅35 (1⋅22, 1⋅50)**	**1⋅18 (1⋅06, 1⋅31)**	623 (14)	**1⋅92 (1⋅64, 2⋅26)**	**1⋅39 (1⋅18, 1⋅64)**
*P* for ACE trend		**<0⋅0001**	**<0⋅0001**		**0⋅0003**	**0⋅0033**		**<0⋅0001**	**0⋅0308**		**<0⋅0001**	**0⋅0029**		**<0⋅0001**	**<0⋅0001**
By ACE category															
Abuse															
None	22 454 (73)	1⋅00 (ref)	1⋅00 (ref)	9466 (75)	1⋅00 (ref)	1⋅00 (ref)	4932 (80)	1⋅00 (ref)	1⋅00 (ref)	4804 (62)	1⋅00 (ref)	1⋅00 (ref)	3252 (74)	1⋅00 (ref)	1⋅00 (ref)
Any	8400 (27)	**1⋅12 (1⋅07, 1⋅17)**	**1⋅06 (1⋅01, 1⋅11)**	3110 (25)	1⋅01 (0⋅94, 1⋅08)	1⋅02 (0⋅95, 1⋅10)	1261 (20)	**1⋅13 (1⋅01, 1⋅26)**	1⋅07 (0⋅95, 1⋅19)	2900 (38)	**1⋅14 (1⋅05, 1⋅24)**	1⋅07 (0⋅98, 1⋅16)	1129 (26)	**1⋅31 (1⋅17, 1⋅48)**	1⋅09 (0⋅97, 1⋅24)
Neglect															
None	24 965 (81)	1⋅00 (ref)	1⋅00 (ref)	10 230 (81)	1⋅00 (ref)	1⋅00 (ref)	5252 (85)	1⋅00 (ref)	1⋅00 (ref)	5753 (75)	1⋅00 (ref)	1⋅00 (ref)	3730 (85)	1⋅00 (ref)	1⋅00 (ref)
Any	5889 (19)	**1⋅20 (1⋅15, 1⋅27)**	**1⋅07 (1⋅02, 1⋅13)**	2346 (19)	**1⋅16 (1⋅07, 1⋅26)**	**1⋅09 (1⋅00, 1⋅18)**	941 (15)	**1⋅16 (1⋅03, 1⋅32)**	1⋅01 (0⋅89, 1⋅14)	1951 (25)	**1⋅18 (1⋅08, 1⋅30)**	1⋅06 (0⋅97, 1⋅16)	651 (15)	**1⋅42 (1⋅22, 1⋅64)**	1⋅13 (0⋅97, 1⋅32)
Household dysfunction															
None	15 377 (50)	1⋅00 (ref)	1⋅00 (ref)	5962 (47)	1⋅00 (ref)	1⋅00 (ref)	3021 (49)	1⋅00 (ref)	1⋅00 (ref)	3808 (49)	1⋅00 (ref)	1⋅00 (ref)	2586 (59)	1⋅00 (ref)	1⋅00 (ref)
Any	15 477 (50)	**1⋅24 (1⋅19, 1⋅29)**	**1⋅16 (1⋅11, 1⋅20)**	6614 (53)	**1⋅13 (1⋅06, 1⋅20)**	**1⋅12 (1⋅05, 1⋅19)**	3172 (51)	**1⋅17 (1⋅07, 1⋅28)**	**1⋅10 (1⋅01, 1⋅21)**	3895 (51)	**1⋅28 (1⋅19, 1⋅39)**	**1⋅18 (1⋅09, 1⋅28)**	1795 (41)	**1⋅58 (1⋅42, 1⋅75)**	**1⋅29 (1⋅16, 1⋅44)**
By individual ACE															
*Abuse*															
Emotional Abuse															
None	25 281 (82)	1⋅00 (ref)	1⋅00 (ref)	10 683 (85)	1⋅00 (ref)	1⋅00 (ref)	5324 (86)	1⋅00 (ref)	1⋅00 (ref)	5735 (74)	1⋅00 (ref)	1⋅00 (ref)	3539 (81)	1⋅00 (ref)	1⋅00 (ref)
Any	5573 (18)	**1⋅16 (1⋅10, 1⋅22)**	1⋅05 (1⋅00, 1⋅11)	1893 (15)	1⋅04 (0⋅95, 1⋅13)	1⋅01 (0⋅93, 1⋅11)	869 (14)	**1⋅17 (1⋅03, 1⋅33)**	1⋅07 (0⋅94, 1⋅22)	1969 (26)	**1⋅19 (1⋅09, 1⋅30)**	1⋅07 (0⋅98, 1⋅18)	842 (19)	**1⋅33 (1⋅17, 1⋅52)**	1⋅07 (0⋅93, 1⋅23)
Physical Abuse															
None	26 052 (84)	1⋅00 (ref)	1⋅00 (ref)	10 865 (86)	1⋅00 (ref)	1⋅00 (ref)	5369 (87)	1⋅00 (ref)	1⋅00 (ref)	6141 (80)	1⋅00 (ref)	1⋅00 (ref)	3677 (84)	1⋅00 (ref)	1⋅00 (ref)
Any	4802 (16)	**1⋅19 (1⋅13, 1⋅26)**	**1⋅07 (1⋅02, 1⋅14)**	1711 (14)	1⋅04 (0⋅95, 1⋅14)	1⋅02 (0⋅93, 1⋅11)	824 (13)	**1⋅18 (1⋅04, 1⋅35)**	1⋅10 (0⋅97, 1⋅26)	1563 (20)	**1⋅23 (1⋅11, 1⋅35)**	1⋅08 (0⋅98, 1⋅20)	704 (16)	**1⋅48 (1⋅28, 1⋅71)**	**1⋅16 (1⋅00, 1⋅35)**
Sexual Abuse															
None	26 451 (86)	1⋅00 (ref)	1⋅00 (ref)	10 758 (86)	1⋅00 (ref)	1⋅00 (ref)	5783 (93)	1⋅00 (ref)	1⋅00 (ref)	5915 (77)	1⋅00 (ref)	1⋅00 (ref)	3995 (91)	1⋅00 (ref)	1⋅00 (ref)
Any	4403 (14)	**1⋅12 (1⋅06, 1⋅18)**	**1⋅09 (1⋅03, 1⋅15)**	1818 (15)	1⋅00 (0⋅92, 1⋅09)	1⋅03 (0⋅94, 1⋅12)	410 (6⋅6)	1⋅03 (0⋅87, 1⋅23)	1⋅02 (0⋅85, 1⋅22)	1789 (23)	**1⋅18 (1⋅08, 1⋅30)**	**1⋅12 (1⋅02, 1⋅23)**	386 (8⋅8)	**1⋅39 (1⋅15, 1⋅67)**	**1⋅25 (1⋅03, 1⋅51)**
*Neglect*															
Emotional Neglect															
None	25 569 (83)	1⋅00 (ref)	1⋅00 (ref)	10 478 (83)	1⋅00 (ref)	1⋅00 (ref)	5421 (88)	1⋅00 (ref)	1⋅00 (ref)	5869 (76)	1⋅00 (ref)	1⋅00 (ref)	3801 (87)	1⋅00 (ref)	1⋅00 (ref)
Any	5285 (17)	**1⋅20 (1⋅14, 1⋅27)**	**1⋅07 (1⋅02, 1⋅13)**	2098 (17)	**1⋅15 (1⋅06, 1⋅25)**	1⋅08 (0⋅99, 1⋅17)	772 (12)	**1⋅22 (1⋅07, 1⋅39)**	1⋅06 (0⋅92, 1⋅21)	1835 (24)	**1⋅17 (1⋅07, 1⋅29)**	1⋅06 (0⋅96, 1⋅16)	580 (13)	**1⋅39 (1⋅19, 1⋅63)**	1⋅13 (0⋅96, 1⋅32)
Physical Neglect															
None	28 650 (93)	1⋅00 (ref)	1⋅00 (ref)	11 750 (93)	1⋅00 (ref)	1⋅00 (ref)	5718 (92)	1⋅00 (ref)	1⋅00 (ref)	7071 (92)	1⋅00 (ref)	1⋅00 (ref)	4111 (94)	1⋅00 (ref)	1⋅00 (ref)
Any	2204 (7⋅1)	**1⋅22 (1⋅13, 1⋅32)**	1⋅07 (0⋅99, 1⋅16)	826 (6⋅6)	1⋅05 (0⋅93, 1⋅19)	0⋅98 (0⋅86, 1⋅11)	475 (7⋅7)	1⋅07 (0⋅91, 1⋅27)	0⋅94 (0⋅79, 1⋅11)	633 (8⋅2)	**1⋅36 (1⋅18, 1⋅57)**	**1⋅20 (1⋅03, 1⋅38)**	270 (6⋅2)	**1⋅66 (1⋅33, 2⋅07)**	**1⋅32 (1⋅05, 1⋅65)**
*Household dysfunction*															
Parents divorced															
None	20 813 (67)	1⋅00 (ref)	1⋅00 (ref)	7970 (63)	1⋅00 (ref)	1⋅00 (ref)	3940 (64)	1⋅00 (ref)	1⋅00 (ref)	5568 (72)	1⋅00 (ref)	1⋅00 (ref)	3335 (76)	1⋅00 (ref)	1⋅00 (ref)
Any	10 041 (33)	**1⋅25 (1⋅20, 1⋅31)**	**1⋅17 (1⋅12, 1⋅22)**	4606 (37)	**1⋅12 (1⋅05, 1⋅19)**	**1⋅10 (1⋅04, 1⋅18)**	2253 (36)	**1⋅11 (1⋅01, 1⋅21)**	1⋅05 (0⋅96, 1⋅16)	2136 (28)	**1⋅40 (1⋅28, 1⋅52)**	**1⋅25 (1⋅14, 1⋅37)**	1046 (24)	**1⋅85 (1⋅63, 2⋅10)**	**1⋅54 (1⋅36, 1⋅76)**
Mother abused															
None	27 228 (88)	1⋅00 (ref)	1⋅00 (ref)	11 023 (88)	1⋅00 (ref)	1⋅00 (ref)	5574 (90)	1⋅00 (ref)	1⋅00 (ref)	6664 (87)	1⋅00 (ref)	1⋅00 (ref)	3967 (91)	1⋅00 (ref)	1⋅00 (ref)
Any	3626 (12)	**1⋅20 (1⋅13, 1⋅28)**	**1⋅12 (1⋅05, 1⋅19)**	1553 (12)	0⋅98 (0⋅90, 1⋅08)	0⋅98 (0⋅89, 1⋅08)	619 (10)	1⋅13 (0⋅97, 1⋅31)	1⋅06 (0⋅91, 1⋅23)	1040 (13)	**1⋅46 (1⋅30, 1⋅64)**	**1⋅33 (1⋅18, 1⋅49)**	414 (9⋅4)	**1⋅67 (1⋅39, 2⋅00)**	**1⋅28 (1⋅07, 1⋅55)**
Live with alcohol or drug abuse															
None	23 820 (77)	1⋅00 (ref)	1⋅00 (ref)	9950 (79)	1⋅00 (ref)	1⋅00 (ref)	4951 (80)	1⋅00 (ref)	1⋅00 (ref)	5511 (72)	1⋅00 (ref)	1⋅00 (ref)	3408 (78)	1⋅00 (ref)	1⋅00 (ref)
Any	7034 (23)	**1⋅19 (1⋅14, 1⋅25)**	**1⋅13 (1⋅07, 1⋅18)**	2626 (21)	**1⋅08 (1⋅00, 1⋅17)**	1⋅08 (1⋅00, 1⋅16)	1242 (20)	**1⋅20 (1⋅07, 1⋅34)**	**1⋅14 (1⋅02, 1⋅27)**	2193 (28)	**1⋅23 (1⋅12, 1⋅34)**	**1⋅15 (1⋅05, 1⋅25)**	973 (22)	**1⋅38 (1⋅22, 1⋅57)**	**1⋅19 (1⋅05, 1⋅35)**
Live with depression/suicide															
None	26 672 (86)	1⋅00 (ref)	1⋅00 (ref)	11 171 (89)	1⋅00 (ref)	1⋅00 (ref)	5657 (91)	1⋅00 (ref)	1⋅00 (ref)	6043 (78)	1⋅00 (ref)	1⋅00 (ref)	3801 (87)	1⋅00 (ref)	1⋅00 (ref)
Any	4182 (14)	**1⋅17 (1⋅10, 1⋅24)**	**1⋅10 (1⋅03, 1⋅16)**	1405 (11)	**1⋅12 (1⋅02, 1⋅24)**	1⋅09 (0⋅99, 1⋅21)	536 (8⋅7)	**1⋅34 (1⋅14, 1⋅57)**	**1⋅19 (1⋅01, 1⋅39)**	1661 (22)	1⋅08 (0⋅98, 1⋅18)	1⋅04 (0⋅95, 1⋅15)	580 (13)	**1⋅32 (1⋅13, 1⋅54)**	1⋅11 (0⋅94, 1⋅30)
Family member in prison															
None	27 439 (89)	1⋅00 (ref)	1⋅00 (ref)	10 908 (87)	1⋅00 (ref)	1⋅00 (ref)	5230 (84)	1⋅00 (ref)	1⋅00 (ref)	7201 (94)	1⋅00 (ref)	1⋅00 (ref)	4100 (94)	1⋅00 (ref)	1⋅00 (ref)
Any	3415 (11)	**1⋅34 (1⋅25, 1⋅42)**	**1⋅21 (1⋅13, 1⋅29)**	1668 (13)	**1⋅27 (1⋅16, 1⋅39)**	**1⋅21 (1⋅10, 1⋅32)**	963 (16)	**1⋅30 (1⋅15, 1⋅47)**	**1⋅17 (1⋅04, 1⋅33)**	503 (6⋅5)	**1⋅46 (1⋅25, 1⋅72)**	**1⋅27 (1⋅08, 1⋅50)**	281 (6⋅4)	**1⋅58 (1⋅27, 1⋅96)**	1⋅16 (0⋅93, 1⋅44)

1 Adjustment for income is in four categories (<$15 000; $15 000–<$25 000; $25 000–<$50 000; ≥ $50 000). The bold indicates statistically significant result at P<0.05

Reporting any ACE was associated broadly with the HEI-10 components (Table 4). In fact, every HEI-10 component was associated with any ACE in at least one race/sex group. Among all, every adequacy component was associated with any ACE except total protein foods. The strongest of these findings include a 26 % increase in odds of insufficient total fruit intake (OR 1⋅26; 95 % CI 1⋅21, 1⋅32), a 24 % increase in odds of insufficient whole fruit intake (OR 1⋅24; 95 % CI 1⋅19, 1⋅29) and a 13 % increase in odds of insufficient total vegetable intake (OR 1⋅13; 95 % CI 1⋅09, 1⋅18). Similarly, two of the three moderation components were associated with any ACE, namely solid fat/alcohol/sugar for an 18 % increase in odds of unhealthy excess (OR 1⋅18; 95 % CI 1⋅14, 1⋅23) and refined grains for a 4 % decrease in odds of unhealthy excess (OR 0⋅96; 95 % CI 0⋅92, 1⋅00).

**Table 4.** Association of adverse childhood experiences (ACEs) with HEI components

	All			Black Females			Black Males			White Females			White Males		
	*n* (%)	Race and sex adjusted OR	Race, sex and income adjusted OR^1^	*n* (%)	Unadjusted OR	Income adjusted OR^1^	*n* (%)	Unadjusted OR	Income adjusted OR^1^	*n* (%)	Unadjusted OR	Income adjusted OR^1^	*n* (%)	Unadjusted OR	Income adjusted OR^1^
*Adequacy Components*															
Total Fruit															
No ACE	13 150 (43)	1⋅00 (ref)	1⋅00 (ref)	5188 (41)	1⋅00 (ref)	1⋅00 (ref)	2707 (44)	1⋅00 (ref)	1⋅00 (ref)	3030 (39)	1⋅00 (ref)	1⋅00 (ref)	2225 (51)	1⋅00 (ref)	1⋅00 (ref)
Any ACE	17 704 (57)	**1⋅26 (1⋅21, 1⋅32)**	**1⋅22 (1⋅17, 1⋅27)**	7388 (59)	**1⋅24 (1⋅16, 1⋅33)**	**1⋅24 (1⋅16, 1⋅32)**	3486 (56)	**1⋅21 (1⋅10, 1⋅32)**	**1⋅15 (1⋅05, 1⋅26)**	4674 (61)	**1⋅26 (1⋅16, 1⋅36)**	**1⋅22 (1⋅12, 1⋅32)**	2156 (49)	**1⋅43 (1⋅29, 1⋅59)**	**1⋅26 (1⋅12, 1⋅40)**
Whole Fruit															
By Any ACE															
None	13 150 (43)	1⋅00 (ref)	1⋅00 (ref)	5188 (41)	1⋅00 (ref)	1⋅00 (ref)	2707 (44)	1⋅00 (ref)	1⋅00 (ref)	3030 (39)	1⋅00 (ref)	1⋅00 (ref)	2225 (51)	1⋅00 (ref)	1⋅00 (ref)
Any	17 704 (57)	**1⋅24 1⋅19, 1⋅29)**	**1⋅19 (1⋅14, 1⋅24)**	7388 (59)	**1⋅24 (1⋅16, 1⋅32)**	**1⋅23 (1⋅15, 1⋅32)**	3486 (56)	**1⋅19 (1⋅09, 1⋅30)**	**1⋅14 (1⋅04, 1⋅25)**	4674 (61)	**1⋅24 (1⋅14, 1⋅35)**	**1⋅21 (1⋅11, 1⋅32)**	2156 (49)	**1⋅31 (1⋅18, 1⋅46)**	**1⋅15 (1⋅03, 1⋅28)**
Whole Grains															
By Any ACE															
None	13 150 (43)	1⋅00 (ref)	1⋅00 (ref)	5188 (41)	1⋅00 (ref)	1⋅00 (ref)	2707 (44)	1⋅00 (ref)	1⋅00 (ref)	3030 (39)	1⋅00 (ref)	1⋅00 (ref)	2225 (51)	1⋅00 (ref)	1⋅00 (ref)
Any	17 704 (57)	**1⋅09 (1⋅04, 1⋅13)**	1⋅04 (1⋅00, 1⋅08)	7388 (59)	**1⋅09 (1⋅02, 1⋅16)**	**1⋅08 (1⋅01, 1⋅15)**	3486 (56)	0⋅97 (0⋅89, 1⋅06)	0⋅92 (0⋅84, 1⋅01)	4674 (61)	**1⋅10 (1⋅02, 1⋅19)**	1⋅04 (0⋅96, 1⋅13)	2156 (49)	**1⋅21 (1⋅09, 1⋅34)**	1⋅08 (0⋅97, 1⋅21)
Total Dairy Products															
By Any ACE															
None	13 150 (43)	1⋅00 (ref)	1⋅00 (ref)	5188 (41)	1⋅00 (ref)	1⋅00 (ref)	2707 (44)	1⋅00 (ref)	1⋅00 (ref)	3030 (39)	1⋅00 (ref)	1⋅00 (ref)	2225 (51)	1⋅00 (ref)	1⋅00 (ref)
Any	17 704 (57)	**1⋅09 (1⋅04, 1⋅13)**	**1⋅05 (1⋅01, 1⋅10)**	7388 (59)	1⋅04 (0⋅98, 1⋅11)	1⋅03 (0⋅96, 1⋅09)	3486 (56)	**1⋅16 (1⋅06, 1⋅27)**	**1⋅15 (1⋅05, 1⋅26)**	4674 (61)	1⋅08 (1⋅00, 1⋅18)	1⋅04 (0⋅95, 1⋅13)	2156 (49)	**1⋅14 (1⋅00, 1⋅24)**	1⋅03 (0⋅92, 1⋅15)
Total Vegetables															
By Any ACE															
None	13 150 (43)	1⋅00 (ref)	1⋅00 (ref)	5188 (41)	1⋅00 (ref)	1⋅00 (ref)	2707 (44)	1⋅00 (ref)	1⋅00 (ref)	3030 (39)	1⋅00 (ref)	1⋅00 (ref)	2225 (51)	1⋅00 (ref)	1⋅00 (ref)
Any	17 704 (57)	**1⋅13 (1⋅09, 1⋅18)**	**1⋅09 (1⋅05, 1⋅14)**	7388 (59)	**1⋅09 (1⋅02, 1⋅16)**	**1⋅09 (1⋅02, 1⋅16)**	3486 (56)	**1⋅18 (1⋅08, 1⋅29)**	**1⋅14 (1⋅04, 1⋅25)**	4674 (61)	**1⋅14 (1⋅05, 1⋅24)**	**1⋅10 (1⋅01, 1⋅19)**	2156 (49)	**1⋅16 (1⋅05, 1⋅29)**	1⋅07 (0⋅96, 1⋅19)
Greens and Beans															
By Any ACE															
None	13 150 (43)	1⋅00 (ref)	1⋅00 (ref)	5188 (41)	1⋅00 (ref)	1⋅00 (ref)	2707 (44)	1⋅00 (ref)	1⋅00 (ref)	3030 (39)	1⋅00 (ref)	1⋅00 (ref)	2225 (51)	1⋅00 (ref)	1⋅00 (ref)
Any	17 704 (57)	**1⋅09 (1⋅04, 1⋅14)**	**1⋅06 (1⋅02, 1⋅11)**	7388 (59)	**1⋅11 (1⋅04, 1⋅19)**	**1⋅12 (1⋅04, 1⋅20)**	3486 (56)	**1⋅20 (1⋅09, 1⋅31)**	**1⋅16 (1⋅05, 1⋅27)**	4674 (61)	1⋅00 (0⋅92, 1⋅09)	0⋅98 (0⋅90, 1⋅06)	2156 (49)	1⋅05 (0⋅95, 1⋅17)	1⋅01 (0⋅91, 1⋅13)
Total Protein Foods															
By Any ACE															
None	13 150 (43)	1⋅00 (ref)	1⋅00 (ref)	5188 (41)	1⋅00 (ref)	1⋅00 (ref)	2707 (44)	1⋅00 (ref)	1⋅00 (ref)	3030 (39)	1⋅00 (ref)	1⋅00 (ref)	2225 (51)	1⋅00 (ref)	1⋅00 (ref)
Any	17 704 (57)	0⋅97 (0⋅92, 1⋅02)	0⋅96 (0⋅91, 1⋅01)	7388 (59)	0⋅93 (0⋅86, 1⋅01)	0⋅93 (0⋅85, 1⋅01)	3486 (56)	**1⋅18 (1⋅04, 1⋅34)**	**1⋅18 (1⋅04, 1⋅33)**	4674 (61)	0⋅93 (0⋅84, 1⋅03)	**0⋅90 (0⋅82, 1⋅00)**	2156 (49)	0⋅95 (0⋅83, 1⋅08)	0⋅92 (0⋅80, 1⋅06)
Seafood and Plant Proteins															
By Any ACE															
None	13 150 (43)	1⋅00 (ref)	1⋅00 (ref)	5188 (41)	1⋅00 (ref)	1⋅00 (ref)	2707 (44)	1⋅00 (ref)	1⋅00 (ref)	3030 (39)	1⋅00 (ref)	1⋅00 (ref)	2225 (51)	1⋅00 (ref)	1⋅00 (ref)
Any	17 704 (57)	**1⋅08 (1⋅04, 1⋅13)**	1⋅03 (0⋅98, 1⋅07)	7388 (59)	0⋅99 (0⋅93, 1⋅06)	0⋅99 (0⋅92, 1⋅06)	3486 (56)	**1⋅14 (1⋅03, 1⋅25)**	1⋅10 (1⋅00, 1⋅21)	4674 (61)	**1⋅10 (1⋅01, 1⋅20)**	1⋅02 (0⋅94, 1⋅11)	2156 (49)	**1⋅23 (1⋅10, 1⋅37)**	1⋅05 (0⋅93, 1⋅18)
Fatty Acids (ratio of poly- and monounsaturated to saturated)															
By Any ACE															
None	13 150 (43)	1⋅00 (ref)	1⋅00 (ref)	5188 (41)	1⋅00 (ref)	1⋅00 (ref)	2707 (44)	1⋅00 (ref)	1⋅00 (ref)	3030 (39)	1⋅00 (ref)	1⋅00 (ref)	2225 (51)	1⋅00 (ref)	1⋅00 (ref)
Any	17 704 (57)	**1⋅11 (1⋅06, 1⋅15)**	**1⋅08 (1⋅03, 1⋅12)**	7388 (59)	1⋅06 (1⋅00, 1⋅13)	1⋅06 (1⋅00, 1⋅13)	3486 (56)	1⋅04 (0⋅95, 1⋅13)	1⋅01 (0⋅92, 1⋅10)	4674 (61)	**1⋅16 (1⋅07, 1⋅26)**	**1⋅14 (1⋅05, 1⋅24)**	2156 (49)	**1⋅18 (1⋅06, 1⋅31)**	1⋅10 (0⋅98, 1⋅22)
*Moderation Components*															
Sodium															
By Any ACE															
None	13 150 (43)	1⋅00 (ref)	1⋅00 (ref)	5188 (41)	1⋅00 (ref)	1⋅00 (ref)	2707 (44)	1⋅00 (ref)	1⋅00 (ref)	3030 (39)	1⋅00 (ref)	1⋅00 (ref)	2225 (51)	1⋅00 (ref)	1⋅00 (ref)
Any	17 704 (57)	1⋅02 (0⋅98, 1⋅06)	1⋅00 (0⋅96, 1⋅04)	7388 (59)	0⋅98 (0⋅92, 1⋅04)	0⋅97 (0⋅91, 1⋅03)	3486 (56)	0⋅98 (0⋅90, 1⋅07)	0⋅97 (0⋅89, 1⋅06)	4674 (61)	1⋅01 (0⋅93, 1⋅09)	0⋅99 (0⋅91, 1⋅07)	2156 (49)	**1⋅21 (1⋅09, 1⋅35)**	1⋅10 (0⋅99, 1⋅23)
Solid fat, alcohol, added sugar															
By Any ACE															
None	13 150 (43)	1⋅00 (ref)	1⋅00 (ref)	5188 (41)	1⋅00 (ref)	1⋅00 (ref)	2707 (44)	1⋅00 (ref)	1⋅00 (ref)	3030 (39)	1⋅00 (ref)	1⋅00 (ref)	2225 (51)	1⋅00 (ref)	1⋅00 (ref)
Any	17 704 (57)	**1⋅18 (1⋅14, 1⋅23)**	**1⋅14 (1⋅09, 1⋅18)**	7388 (59)	**1⋅12 (1⋅06, 1⋅20)**	**1⋅12 (1⋅05, 1⋅19)**	3486 (56)	**1⋅13 (1⋅03, 1⋅23)**	1⋅09 (1⋅00, 1⋅20)	4674 (61)	**1⋅20 (1⋅11, 1⋅31)**	**1⋅14 (1⋅05, 1⋅24)**	2156 (49)	**1⋅37 (1⋅24, 1⋅53)**	**1⋅21 (1⋅09, 1⋅35)**
Refined Grains															
By Any ACE															
None	13 150 (43)	1⋅00 (ref)	1⋅00 (ref)	5188 (41)	1⋅00 (ref)	1⋅00 (ref)	2707 (44)	1⋅00 (ref)	1⋅00 (ref)	3030 (39)	1⋅00 (ref)	1⋅00 (ref)	2225 (51)	1⋅00 (ref)	1⋅00 (ref)
Any	17 704 (57)	**0⋅96 (0⋅92, 1⋅00)**	**0⋅92 (0⋅88, 0⋅95)**	7388 (59)	**0⋅90 (0⋅85, 0⋅96)**	**0⋅89 (0⋅84, 0⋅95)**	3486 (56)	0⋅98 (0⋅89, 1⋅07)	0⋅96 (0⋅88, 1⋅05)	4674 (61)	0⋅94 (0⋅87, 1⋅02)	**0⋅88 (0⋅81, 0⋅96)**	2156 (49)	**1⋅12 (1⋅01, 1⋅25)**	0⋅99 (0⋅89, 1⋅10)

1 Adjustment for income is in four categories (<$15 000; $15 000–<$25 000; $25 000–<$50 000; ≥ $50 000). The bold indicates statistically significant result at P<0.05

For the secondary analysis adjusting for income, the associations among all remained in every component except whole grains and seafood and plant proteins. Furthermore, the components with the strongest associations before adjustment remained the strongest after adjustment, with adjusted ORs of 1⋅22 for total fruit (95 % CI 1⋅17, 1⋅27), 1⋅19 for whole fruit (95 % CI 1⋅14, 1⋅24) and 1⋅14 for solid fat/alcohol/added sugar (95 % CI 1⋅09, 1⋅18). These components were notably consistent across the race/sex groupings. For solid fat/alcohol/added sugar, ORs were significant in every case except after income adjustment for Black males. Additionally, we found there to be some race/sex-specific associations, the most notable being increased odds of insufficient greens and beans intake among Blacks (Black females, OR 1⋅11; 95 % CI 1⋅04, 1⋅19; Black males, OR 1⋅20; 95 % CI 1⋅09, 1⋅31) but not among Whites.

## Discussion

Broadly, our findings demonstrate a positive association between ACEs, including any ACE, and worse adult diet quality. This is true for both Blacks and Whites, whether male or female, and is supported by the literature which describes ACEs as having a pervasive association with unhealthy behaviours and poor health across diverse populations^(43)^. Even further, our findings by number of ACEs suggest a dose-response effect. This suggests that the relationship found between ACEs and HEI-10 is causal. Nevertheless, our findings also indicate that the strength of the ACE-HEI association varies by race/sex group. This is consistent with the literature for the relationship between ACEs and other health outcomes^(12,44)^, and indicates that there may be additional factors at play within specific population demographics that need to be addressed in order to effectively estimate the ACE-HEI association in all subgroups.

Of particular interest are the strong associations found between household dysfunction ACEs and HEI-10. We believe that this may be in part due to the more objective aspect of the household dysfunction ACEs (compared to abuse or neglect ACEs), which include divorced parents, physically abused mother, living with alcohol or drug abuser, living with someone with mental illness and having a family member in prison. If this is the case, this also suggests that abuse and neglect ACEs may be underreported^(45,46)^, which would reduce our ability to detect an association between abuse and neglect ACEs with HEI-10. Another possibility is that household dysfunction ACEs truly are more likely to affect the dietary patterns experienced and learned by an individual as a child than abuse or neglect ACEs.

The strong connection between household functioning and health is supported by a study of 280 dyads in Miami, which found associations between self-report of parent–adolescent family functioning and obesity-related behaviours, specifically physical inactivity and poor diet, among Hispanic adolescents^(47)^. Parent–adolescent discrepancy scores in family functioning were found to be negatively associated with both physically active days (*β* −0⋅14; 95 % CI −0⋅26, −0⋅05; *P* < 0⋅05) and fruit/vegetable intake (*β* −0⋅022; 95 % CI −0⋅38, −0⋅09; *P* < 0⋅001). Although the family functioning indicators (positive parenting, parental involvement, family communication, parental monitoring of peers and parent–adolescent communication) used by Lebron *et al.* were distinct from the household dysfunction ACEs, the findings on family dynamics and fruit/vegetable intake are consistent with the present study^(47)^. Additionally, a recent cross-sectional study of 2939 adults using the 2017 Nevada Behavioral Risk Factor Surveillance System further supports our findings between household dysfunction, specifically parental divorce, and diet quality^(48)^. In particular, having three or more ACEs (OR 1⋅42; 95 % CI 1⋅02, 2⋅00) and experiencing parental divorce or separation (OR 1⋅50; 95 % CI 1⋅13, 1⋅98) were found to be associated with lower consumption of fruit and vegetables^(48)^.

Even further, the finding in the present study of household functioning and fruit/vegetable intake has been described internationally. In one recent study of 24 271 older adults in Japan, a sex-stratified multilevel Poisson regression found an association between ACEs and low fruit and vegetable intake that was more pronounced among females (1 ACE, OR 1⋅18; 95 % CI 1⋅07, 1⋅30; 2+ ACEs, OR 1⋅64; 95 % CI 1⋅42, 1⋅89) than males (1 ACE, OR 1⋅17; 95 % CI 1⋅08, 1⋅27; 2+ ACEs, OR 1⋅34; 95 % CI 1⋅20, 1⋅50)^(49)^. Additionally, a study of 11 243 individuals in England by Russell *et al.* using the North West Mental Well-Being Survey found that adults with unhappy and violent childhoods had lower daily fruit and vegetable intake than adults with happy and non-violent childhoods (OR 2⋅67; 95 % CI 2⋅15, 3⋅06; *P* < 0⋅001). However, none of these studies were conducted in a largely low socioeconomic or racial/ethnic minority population^(50)^.

In the present study, many ACE-HEI associations were particularly strong for White males. The reason for this is unclear. However, we suspect other factors are at play among marginalised groups which may have somewhat diminished our ability to detect the ACE-HEI association among non-White males. For instance, race/sex variations in resilience, exposure to other adverse experiences, and/or under-reporting may play a role. These variations could be specific to the largely low-income and Southern context of this population, or more generalisable by race and sex. Future research should explore these possibilities which may help explain the differences in the strength of the ACE-HEI associations by race and sex.

Importantly, there are likely a multitude of causal mechanisms through which ACE exposure leads to poor diet quality. We believe most of these pathways include neurobiological consequences of childhood trauma, where various neuroendocrine, neurochemical and neuroanatomic feature responses may all come into play^(51–56)^. The precise configuration of these responses could vary by the type of ACE, possibly even leading to different manifestations of poor adult diet quality (e.g. adequacy *v.* moderation components)^(57)^. Future studies are needed to explore potential pathways from childhood trauma to poor adult diet quality on the neurobiological level.

The present study suggests the need for interventions that can disrupt the pathway from ACEs to poor diet quality, and potentially downstream health outcomes. For example, ACE screening could be used as an important tool for identifying individuals at risk of poor diet quality and related health issues. Targeting individuals, households and/or communities with higher prevalence of ACEs, especially household dysfunction ACEs, could enhance the efficacy of dietary interventions and reduce disparities in ACE-related outcomes. In addition, interventions aimed at reducing the impact of traumatic experiences have the potential of positively impacting diet quality. Some examples of such interventions include Trauma-Focused Cognitive Behavioral Therapy (TF-CBT), Child-Parent Psychotherapy (CPP) and Eye Movement Desensitization and Reprocessing (EMDR)^(58–61)^. On the structural level, trauma-informed care approaches should also be adopted and trauma-specific services be made available and affordable^(62–67)^. Importantly, the negative outcomes of childhood trauma appear throughout the lifespan, so it may never be too late to intervene. In fact, intervention with adults who were traumatised as children may not only improve health outcomes but decrease intergenerational transmission of trauma as well^(2)^.

To note, there are some limitations to the present study. These include a variety of factors that would have been useful to consider. For instance, we did not have the ages at which the ACEs occurred. Such detailed data would have been helpful since trauma tends to have a greater impact on younger children than older children^(68)^. Secondly, we did not have any information regarding childhood or young adult diet quality. This would be useful to explore when and why individuals with ACEs tend to develop worse diet quality. Similarly, household income as a child would have been useful to further validate the ACE-HEI association independent of income, rather than just adult household income as used in the present study. Where available, future studies should include these and other childhood lifestyle and environmental factors to consider the possibility of confounding, which could not be addressed by the present study. In addition, ACEs do not screen for all potentially traumatic childhood experiences (e.g. bullying, natural disaster, parent death), and HEI-10 may not account for all relevant adult dietary patterns (e.g. eating behaviours). Future studies using other or more extensive methods to estimate diet quality and childhood trauma would, thus, be useful to better understand the breadth of the associations described in the present paper. For instance, interviewing participants as well as their friends or family members could help better estimate childhood trauma and diet quality. This includes ACE severity and duration, which was not considered in the present study. Additionally, food diaries, resilience screening, and more prospective data collection (ACEs, HEI, household income, etc.) throughout childhood and adulthood may be valuable, as well as screening for adult adverse experiences. Health-related variables such as chronic disease, mental disorder, physical activity and sleep quality should also be considered for their potential role within or effect modification of the ACE-HEI pathway, although only socio-demographic and lifestyle variables were included in the study at hand.

Furthermore, the present study utilised self-report data to estimate both HEI-10 and ACE scores. It is possible that there is recall and reporting bias to our findings as a result, but whether this would bias our results towards or away from the null is not clear. Additionally, since participants were excluded who did not return for the second SCCS follow-up questionnaire (*n* 37 774), there may be a healthy worker selection bias to our findings which would bias our results towards the null. The included participants still represent an overall low SES population (as 46 % of the participants in the present study have an annual household income of less than $15 000), but as mentioned earlier the representation of the very lowest socioeconomic position is consequently less strong. Our results could similarly be biased towards the null by the smaller exclusion of participants (*n* 5715) who returned for the second SCCS follow-up questionnaire but did not complete one or more ACE questions within it, since such omissions may reflect sensitivity to the ACE questions.

There are other potential complications to our study as well, which may make our findings more difficult to interpret. For instance, there is research suggesting that poor childhood diet could make children more susceptible to the neurobiological impacts of trauma, as well as research suggesting that good diet quality may help mitigate the impact of trauma^(69,70)^. As a result, the associations found with poor diet quality could reflect a reporting bias where individuals with poorer adult diet quality are more likely to qualify their childhood experiences as significant or traumatic depending on the pervasiveness of their experience determined by childhood or adult diet quality. Additionally, there is research suggesting women with ACEs who consequently develop a dysregulated HPA axis can pass on or ‘transmit’ HPA dysregulation to their child during pregnancy, and that a high-quality diet during pregnancy can mitigate this impact by positively modifying the stress response through dietary factors such as prenatal maternal choline intake^(71)^. Taking this mechanism of intergenerational trauma into account, the trauma and even diet of our participants’ birth mothers could be in part responsible for participant adult dietary quality in the present study. This effect could be compounded after birth through other factors such as emotional feeding practices, family stress internalisation and parental food purchasing behaviours^(28,72)^. Thus, the ACE-HEI association found could reflect not only the trauma experienced by our participants themselves, but the trauma passed down *in utero* or during childhood. This, however, also raises the possibility of a diminished ability to detect association in the present study, since it is possible for an individual impacted by generational trauma to have avoided the traumatic childhood experiences screened for while still being adversely impacted by trauma throughout their life (e.g. diet quality). Nevertheless, the implications of our study remain the same whether or not these phenomena play an explanatory role in our findings: diet quality should be paid greater attention to for people who have experienced childhood trauma, trauma interventions could be useful to improve poor diet quality, and efforts to intercede in the ACE-HEI pathway could reduce health disparities, disease and mortality rates.

The present study had notable strengths as well. First, the large sample size of 30 854 participants supports the precision of the association found between ACEs and HEI-10. Second, this large sample size, particularly among Blacks, allowed us to conduct race/sex-specific analyses, finding both universal prevalence of the association and consistent manifestations by race and sex. Third, there were notable strengths regarding questionnaire design. The FFQ utilised by the SCCS was designed to account for the race and geographic region, and most SCCS participants completed the FFQ through an in-person computer-assisted interview. Telephone administration was offered for the second follow-up questionnaire which included the ACE questionnaire, and the ACE questionnaire that was utilised modelled the standardised framework for ACE assessment. Furthermore, the questions on the ACE questionnaire used were succinct to prevent burnout, but also highly specific to maximise objectivity, aid validity and prevent misclassification bias^(73)^. Moreover, a comprehensive dietary assessment was used to analyze the relationship between ACEs and diet quality, which no other study has done to date. In addition, component analysis in the present study supports the conclusion that ACEs are broadly associated with poor adult diet quality. Lastly, the present study allowed us to examine the association of ACEs and HEI-10 independent of household income, which could play a significant role due to the high cost of a high-quality diet^(74)^.

In conclusion, in this large study of primarily low-income individuals in the southeastern US, having had any ACE was associated with an increased odds of a worse diet quality as an adult. This association was consistent among Blacks and Whites, and males and females, and became stronger with increasing number of ACEs. Moreover, adjusting for adult SES, as estimated by household income, did not remove the association. As diet quality is directly linked to health outcomes, our study suggests that further research is needed to understand the mechanisms through which ACEs increase the likelihood of poor adult diet, and thus the potential for dietary and therapeutic interventions to improve health outcomes among individuals who have experienced childhood trauma.
